# Assessing urban resilience based on production-living-ecological system using degree of coupling coordination: A case of Sichuan

**DOI:** 10.1371/journal.pone.0304002

**Published:** 2024-05-23

**Authors:** Huan Shi, Yuan Hu, Lu Gan

**Affiliations:** 1 Sichuan Emergency Management College, School of Emergency Management, Xihua University, Chengdu, Sichuan Province, China; 2 College of Management, Sichuan Center for Rural Development Research, Sichuan Agricultural University, Chengdu, Sichuan Province, China; 3 College of Architecture and Urban-Rural Planning, Sichuan Agricultural University, Dujiangyan, Sichuan Province, China; King Khalid University, SAUDI ARABIA

## Abstract

The issue of urban resilience plays great significance and value for the sustainable development of cities, which has attracted increasing attention from scholars and governments, especially in the western region of China. Based on the Production-Living-Ecological (PLE) system, this study attempts to describe urban resilience by the combination system that contains with P,L,E subsystem. The integrated approach including FAHP-EM,GRA-TOPSIS, CCDM, and ODM is proposed to reveal the urban resilience level and seek out the key constraints’ indicators. Then, an empirical analysis of panel data of 18 cities in Sichuan Province from 2011 to 2021 was conducted to analyze the development process. The valuation results suggested that:(1)for urban resilience level, most cities at the moderate imbalance level and basically maintained at this level, only Chengdu is reaching the basic coordination level since in 2013.(2)The insufficient development of P,L,E subsystem is the reason for the moderate imbalance development, especially the key limiting factor is the P subsystem’s low development level.(3)the most prominent obstacle indicators are x1(per capita local financial expenditure on science and technology), x2(per capita of R&D spending), x8(total export-import per capita), x14(number of people with basic medical insurance), x22(length of urban drainage pipeline), x23(number of public toilets per person) and the contribution values reach 7.56%,7.49%,11.02%, 9.14%,12.53%, 12.60% respectively. The detailed reference suggestions and effective measures put forwarded for policy makers and planners to promote urban resilience in Western China.

## 1 Introduction

Evaluating urban resilience has become important in urban sustainable development, especially in against disasters and unexpected events [1]. The increasing disasters such as cyclone or fire, flood, flu or influx of refugees, have caused huge casualties and irreparable economic losses since 1980 [2]. Urban resilience plays an important role in preparing for unexpected events, especially in major natural disasters and emergencies environment extreme and other conditions, and its can exert advantages the ability of a city to resist disasters, reduce disaster losses, and allocate resources reasonably to quickly recover from disasters [3]. With the outbreak of the global COVID-19 pandemic, the emergence of the COVID-19 has threatened many industries and forced cities to reassess and solve their urban resilience problems [4].

In recent years, scholars have paid increasing attention to urban resilience and conducted a variety of studies, but there has limited efforts on the quantitative evaluation of urban resilience [5]. Simultaneously, the resilience of cities urgently needs improvement because of the frequency and severity of disasters, and it has become an important factor restricting local social and economic development in some regions of China, especially in western areas [6]. Therefore, practical and quantitative scientific urban resilience evaluation research on cities becomes a new valuable research hotspot, as well as specific suggestions need to be further explored. Under these urgent situations, the urban resilience level needs to be evaluated efficiently and utilized further to promote sustainable development in western China.

This study aims to analyze the coupling coordination degree of urban resilience, which plays a critical role in sustainable urban development especially in disaster prevention. However, urban resilience is a complex giant system that aims to achieve PLE system sustainable development in urbanization [7]. Therefore, this paper selects the PLE system as the basic structure to describe the urban resilience, which contains production resilience, living resilience, and ecological resilience subsystem. Then, a comprehensive method is proposed to fit the discussed problem, which including: FAHP-EM, GRA-TOPSIS, CCDM, and ODM. This article closely focuses on how to enhance the resilience level of cities and play a more crucial role in achieving sustainable urban development, and the more reliable recommendations proposed by quantitative models can more conveniently assist users in making decisions.

## 2 Literature review

### 2.1 Urban resilience evaluation

Recently, more and more scholars have begun to focus on urban resilience, so as to realizing organizations recover very quickly from disasters through rational allocation of resources [3]. Holling first proposed the concept of resilience in 1973, as a capability of ecological systems manage and deal with risks in suddenly changes, and then it was added to the urban field at the United Nations World Summit on Sustainable Development in 2002. The main research areas of urban resilience including climate change [8], urban planning, urban risks and disaster hazards [9], smart city [10, 11], green infrastructure and urban sustainability [12], and so on [3]. However, scholars have conducted an increasing number and types of research on urban resilience, while investing limited effort in the quantitative assessment of urban resilience [5].

Urban resilience is a complex and massive system that focuses on cities can cope with potential negative impacts, to achieve sustainable development of PLE system in urbanization [13]. Most studies related to urban resilience have laid a solid foundation for resilience theory and its branches, and have proposed supporting arguments [9]. However, urban resilience as the multi-dimensional concept, is still not a unified definition or a consistent set of descriptors [2]. Such as Mou et al. proposed five subsystems, i.e. population, economy, resource, environment, science & technology, and incorporated them into the conceptual framework of urban sustainability and resilience [5]. Zhou et al. put out coupling coordination analysis and use obstacle factors identification of rural living-production-ecological functions [14]. Accordingly, this paper aims to build the urban resilience evaluation system with a multidimensional indicators structure which grouped into three interrelated dimensions: production resilience, living resilience, and ecological resilience.

### 2.2 PLE system

The urban resilience is a complex coupled system can be summed up as integrates production resilience, living resilience, and ecological resilience. Table 1 shows production resilience subsystem have eight indicators, by considering the technological innovation capability [15], the economy of urban [6], and the disaster prevention. In fact, natural disasters often come with huge economic and human casualties [15], therefore, eleven indicators are selected to reflect the pre-disaster readiness capacity and the post-disaster response capacity of social security for the living resilience subsystem. Indicators including urban population [15], social security [6], infrastructure [15] and so on, especially indicator combined with the number of fires and traffic accidents to reflect the urban ability to deal with emergencies. The environmental resilience subsystem selected six indicators to indicate disaster reduction and response capabilities [6, 13, 16].

**Table 1:** The urban resilience evaluation indicator system.

Subsystem	Criterion layer	Primary indicator	Unit	Direction	Justification and effect on urban resilience
Production subsystem (P)	Technological innovation capability	per capita local financial expenditure on science and technology(x1)↑↑	CNY	+	the pre-disaster reduction capacity of science and technology
		per capita of R&D spending (x2)↑	CNY	+	the pre-disaster reduction capacity of technology innovation
	Economy of urban	urban per capita GDP(x3)↑↑	CNY	+	the pre-disaster readiness capacity of economy
		urban per capita disposable income in residents(x4)↑↑	CNY	+	the post-disaster response capacity of disbursing in residents
		per capita general public budget revenue(x5)↑	CNY	+	the post-disaster response capacity of emergency relief disbursing
	Disaster prevention capability	secondary and tertiary industry / GDP(x6)↑↑	%	+	the post-disaster recovery capacity of industry and service
		per capita total retail sales of consumer goods(x7)↑	CNY	+	the post-disaster recovery capacity of consumption
		total export-import per capita(x8)↑	CNY	+	the post-disaster recovery capacity of economy
Living subsystem (L)	Urban population	population density(x9)↑	persons/km^2^	-	the pre-disaster reduction capacity of population
		the number of employees(x10)↑↑	persons	+	the pre-disaster readiness capacity of society
	Social security	per capita local financial expenditure on education(x11)↑	CNY	+	the pre-disaster readiness capacity of the education system
		number of cultural stations(x12)*	piece	+	the pre-disaster readiness capacity of the community assistance
		the number of beds in medical institutions per 10,000-person(x13)↑↑	beds	+	the post-disaster response capacity of the healthcare system
		number of people with basic medical insurance(x14)↑↑	persons	+	the post-disaster response capacity of the medical assistance
		per capita expenditure on social security and employment(x15)↑↑	CNY	+	the post-disaster response capacity of social security
	Infrastructure	number of fires and traffic accidents(x16)*	time	-	the post-disaster response capacity of emergency accidents
		per capita area of roads(x17)↑↑	m^2^	+	the post-disaster response capacity of public infrastructure
		gas coverage rate(x18)↑	%	+	the post-disaster response capacity of living facilities
		water coverage rate(x19)↑	%	+	the post-disaster response capacity of infrastructure
Ecological subsystem (E)	Disaster reduction capacity	green coveraged area as percentage of built districts(x20)↑↑	%	+	the pre-disaster reduction capacity of ecosystem
		per capita park green area(x21)↑↑	m^2^	+	the pre-disaster reduction capacity of ecosystem
	Disaster response capacity	length of urban drainage pipeline(x22)↑	km	+	the post-disaster response capacity of infrastructure
		number of public toilets per person(x23)*	piece	+	The post-disaster response capacity of infrastructure
		sewage treatment rate(x24)↑	%	+	the post-disaster response capacity of living environment
		household garbage treatment rate(x25)↑	%	+	the post-disaster response capacity of living environment

↑↑ denotes the highly cited indicators.

↑ denotes the moderately cited indicators.

* denotes the novel indicators.

This study developed a comprehensive indicator system to evaluate the urban resilience level and its constituent subsystems. Table 1 presents the rationality of the indicators and their impact on urban resilience. The PLE system is used to present urban resilience, which contains production resilience, living resilience, and ecological resilience.

### 2.3 Methods of urban resilience evaluation

Research has shown that many different methods have been used in the field of urban resilience, and analytical methods have been rapidly developing in recent years. The oldest study included Analytic Hierarchy Process (AHP) in the literature on urban resilience analysis, and it is evident that as research progresses, AHP has become the most widely used Multi Criteria Decision Model(MCDM) technology [2]. Some researchers have incorporated Fuzzy set theory or different MCDM methods into their work to promote AHP, such as Grey Relation Analysis (GRA) and Technique for Order Preference by Similarity to Ideal Solution(TOPSIS), in the fields of intelligent cities and urban flood control research [3]. Table 2 shows the comparison of some main evaluation methods for MADM. In addition to MCDM technology, data analysis and statistical methods are also frequently used. Simple methods of statistics are widely used to summarize data obtained from quantitative research and determine possible relationships between variables. Such as factor analysis [17], descriptive analysis [12], sensitivity analysis [18], principal component analysis [19], ANOVA [10], cluster analysis [8], and regression analysis [11].

**Table 2:** Comparison of some main evaluation methods for MADM.

Method	Advantages	Disadvantages
EM [21, 22]	It is easy to use, calculate, and suitable for situations where there is no obvious correlation between evaluation indicators.	It ignores the interaction between indicators and has strong data objectivity.
AHP [2, 3, 22]	It takes into account the interaction between indicators and allows for multi-level evaluation and weight allocation.	It is necessary to establish an accurate judgment matrix for the relationship between indicators, and the determination of weights is more subjective.
GRA [3, 21]	It is able to deal with the non-linear relationship between evaluation indicators, and it is robust to missing or incomplete data	It has high requirements for data, requires a certain amount of data preprocessing capabilities, and the results are sensitive and easily disturbed by data noise.
TOPSIS [2, 3, 21]	It is able to take into account the relative importance and degree of superiority and disadvantage of evaluation indicators	The determination of the weight of the indicator is subjective. There are high requirements for data standardization.

Nowadays, more effective evaluation models are needed to assist decision-makers in developing urban resilience assessment strategies, so as to put forward feasible policy suggestions and development plans. As far as the PLE system is concerned, the Coupling Coordination Degree Model (CCDM) can be used to analyze the coordinated development level of two or more systems, which can clearly explain the content and relationship between subsystems and comprehensive evaluation level [20]. In addition, previous studies have shown that the calculation results of the combined GRA-TOPSIS methods are superior than GRA or TOPSIS method, and are closer to the actual situation [21]. Fuzzy Analytic Hierarchy Process(FAHP) and Entropy Method(EM) are commonly used methods for determining indicator weights, which can help decision-makers to combine subjectivity and objectivity in the problem [22]. Moreover, existing studies have shown that the Obstacle Diagnosis Model((ODM) can calculate the obstacle degree of each evaluation index in the comprehensive evaluation, find out the key factors that restrict the further development of things, and clarify the influence degree of the constraints, so as to provide a scientific basis for formulating scientific and reasonable policies [23]. Consequently, the ODM method is adopted in this paper and calculates the importance of each obstacle factor, and the key factors affect the level of urban resilience could be determined.

### 2.4 Research framework

**Fig 1:**
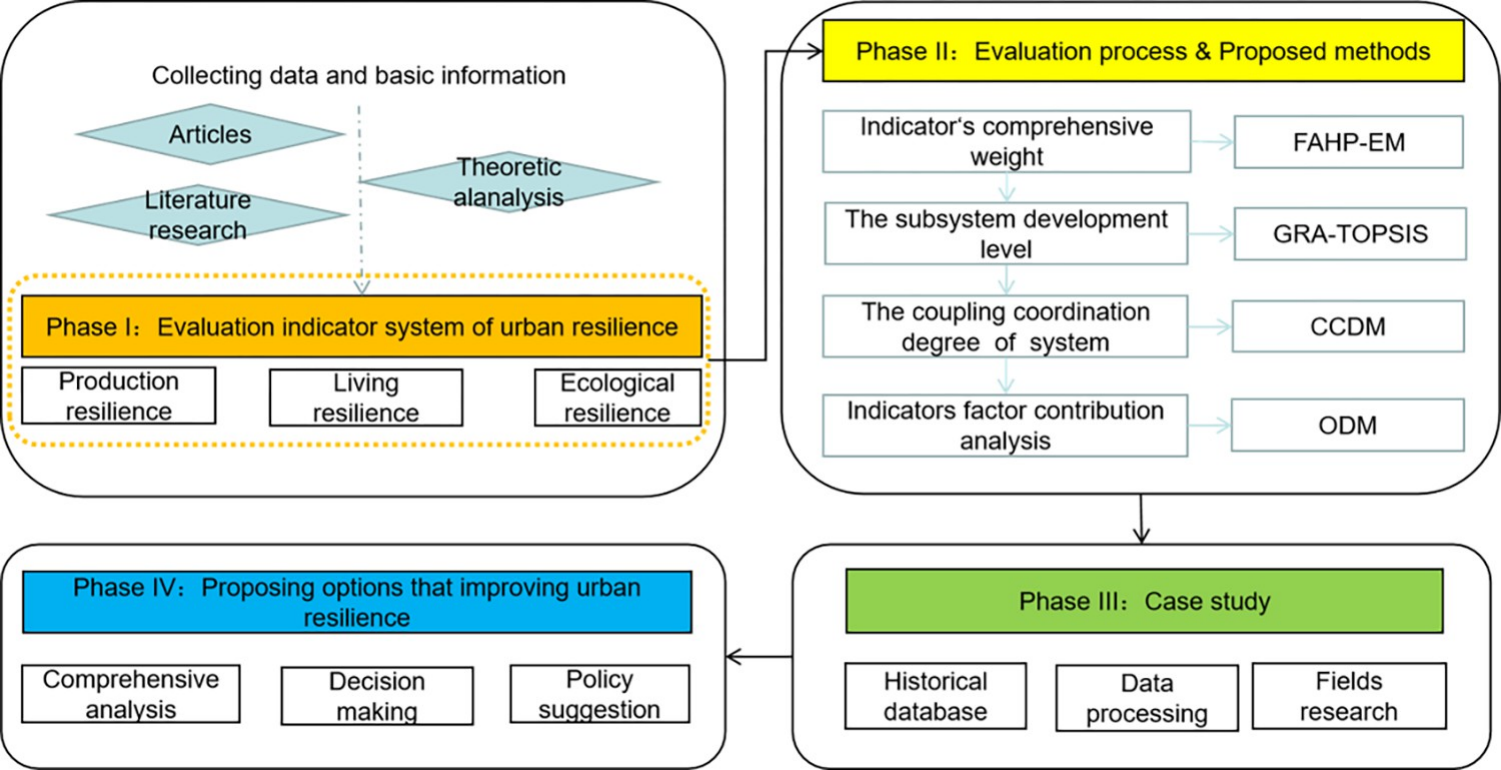
The research framework.

The research framework of urban resilience evaluation can be divided into four phases (Fig 1). Phase I: this study develops an index system that accurately represents urban resilience, which with indicators from production resilience, living resilience, and ecological resilience subsystem; Phase II: the integrated approach including: FAHP-EM, GRA-TOPSIS, CCDM, and ODM is proposed during the urban resilience evaluation process. Phase III: a realistic case study is tested to validate the applicability of the whole approaches, and the urban resilience level for 18 cities is analyzed in Sichuan region during 2011–2021 (most recent available data as of 2023); Phase IV: using ODM to identify key factors that affect their resilience level, some feasible suggestions were proposed for urban resilience in the western region.

## 3 Materials and methods

### 3.1 Methods for evaluating urban resilience level

#### 3.1.1 Normalization

In order to eliminate the effects of different units and dimensions, the collected data is normalized through range methods:

$$y_{ij}=\frac{x_{ij}-min(x_{ij})}{\max\left(x_{ij}\right)-min(x_{ij})}$$ (1)

$$y_{ij}=\frac{\max\left(x_{ij}\right)-x_{ij}}{\max\left(x_{ij}\right)-min(x_{ij})}$$ (2)

Where *x*_*ij*_ is the *j*_*th*_ targeted value of the *i*_*th*_ indicator, whereas *max* (*x*_*ij*_) and *min* (*x*_*ij*_) designate the maximum and minimum values of the indicator among all spatial units, respectively. When the indicator makes positive or negative effects in the system, Eqs (1) and (2) apply, respectively, and then the targeted value after normalization *y*_*ij*_ can be obtained.

#### 3.1.2 Weight calculation. (1) FAHP Process

FAHP can use member functions to handle such ambiguous situations and allow for multiple criteria and simultaneous evaluation. It has been successfully applied to handle complex but fuzzy decision problems [22]. The steps of solving the FAHP are as follows.

Step1 constructing probability matrix: *B =* (*b*_*ij*_)_*m*×*n*_

$$b_{ij}=\frac{l_{ij}+4m_{ij}+u_{ij}}{6}$$ (3)

Where the triangular fuzzy complementary judgment matrix is $R={\left(r_{ij}\right)}_{m\times n};r_{ij}=(l_{ij},m_{ij},u_{ij},)$.

Step2 fuzzy judgment matrix:

$$S=(\begin{array}{cccc} 1 & 1-\frac{u_{12}-l_{12}}{2(l_{12}+m_{12}+u_{12})} & \cdots & 1-\frac{u_{1n}-l_{1n}}{2(l_{1n}+m_{1n}+u_{1n})}\\ 1-\frac{u_{21}-l_{21}}{2(l_{21}+m_{21}+u_{21})} & 1 & \cdots & 1-\frac{u_{1n}-l_{1n}}{2(l_{1n}+m_{1n}+u_{1n})}\\ \vdots & \vdots & \ddots & \vdots \\ 1-\frac{u_{n1}-l_{n1}}{2(l_{n1}+m_{n1}+u_{n1})} & 1-\frac{u_{n2}-l_{n2}}{2(l_{n2}+m_{n2}+u_{n2})} & \cdots \cdots & 1 \end{array})$$ (4)

Step3 adjustment judgment matrix:

$${T=B*S=(b_{ij}*s_{ij})}_{m\times n}$$ (5)

Step4 fuzzy complementary judgment matrix: $\bar{R}$

$${\bar{r}=(t}_{ij}-t_{ji}+1)$$ (6)

Step5 test their consistency:

$$k=p^{1}\dot{+}p^{2}\dot{+}p^{3}\dot{+}\cdots \dot{+}p^{n}$$ (7)

Define elements:

$$p_{ij}=\{\begin{array}{ll} 1 & r_{ij>0.5}\\ 0 & r_{ij}\leq 0.5 \end{array}$$ (8)

Step6 the weight of the indicator:

$$W_{i}=\frac{1}{n}\_\frac{1}{2\alpha }+\frac{1}{n\alpha }\sum_{j=1}^{n}\bar{r_{ij}}(\alpha \geq \frac{n-1}{2})$$ (9)

Step7 hierarchical weighted total ordering:

$$b_{i}=\sum_{j=1}^{m}b_{ij}c_{j}$$ (10)

Test their consistency

$$\rho =\sum_{j=1}^{m}\rho (j)c_{j}$$ (11)

#### (2) Entropy method

Entropy was first introduced into information theory by Claude E. Shannon and has been widely applied in fields such as engineering technology and social economy [24]. Fully utilize the information of the data, calculate based on the changes in information entropy and indicators, and determine the weight of each indicator [25]. The proportion of the indicator:

$$s_{ij}=\frac{1+x_{ij}}{\sum_{i=1}^{n}\left(1+x_{ij}\right)}$$ (12)

*m* elements with *n* indicators, and the information entropy of the indicator can be computed as in the next equation.


$$e_{j}=-\frac{1}{lnm}\sum_{i=1}^{m}s_{ij}lns_{ij}\;(0\leq e_{j}\leq 1)$$ (13)

Entropy redundancy:

$$d_{j}=1-e_{j}$$ (14)

Weight of the indicator:

$$w_{j}=\frac{1-d_{j}}{m-\sum_{j=1}^{n}d_{j}}$$ (15)

#### (3) Blends weights

The game theory method combined with EM and FAHP method is used to get the final weights, and the steps can be referred to the research of Liu et al. [26]. The integrated algorithm integrates the results of subjective and objective to ensure the scientific of the final conclusion [21].

Linear combinations of different vectors:

$$u=\sum_{k=1}^{L}\alpha_{k}u_{k}^{T}\;(\alpha_{k}>0,\sum_{k=1}^{L}\alpha_{k}=1)$$ (16)

Where *u* means a possible weight vector of weight set, *α*_*k*_ stands for linear combination coefficient. Using game theory to optimize L linear combination coefficients *α*_*k*_, minimizing the dispersion between *u* and each *u*_*k*_.


$$min{\left\|\sum_{j=1}^{L}\alpha_{j}u_{j}^{T}-u_{i}\right\|}_{2}(i=1,2\cdots L)$$ (17)

The above equation can be converted into equations:

$$\left[\begin{array}{cccc} u_{1}\cdot u_{1}^{T} & u_{1}\cdot u_{2}^{T} & \cdots & u_{1}\cdot u_{L}^{T}\\ u_{2}\cdot u_{1}^{T} & u_{2}\cdot u_{2}^{T} & \cdots & u_{2}\cdot u_{L}^{T}\\ \vdots & \vdots & \vdots & \vdots \\ u_{L}\cdot u_{1}^{T} & u_{L}\cdot u_{2}^{T} & \cdots & u_{L}\cdot u_{L}^{T} \end{array}][\begin{array}{c} \alpha_{1}\\ \alpha_{2}\\ \vdots \\ \alpha_{L1} \end{array}]=[\begin{array}{c} u_{1}\cdot u_{L}^{T}\\ u_{2}\cdot u_{L}^{T}\\ \vdots \\ u_{L}\cdot u_{L}^{T} \end{array}\right]$$ (18)

Normalized treatment:

$$\alpha_{k}^{*}=\frac{\left|\alpha_{k}\right|}{\sum_{k=1}^{L}\left|\alpha_{k}\right|}$$ (19)

The most satisfied with the comprehensive weight:

$$w=\sum_{k=1}^{L}\alpha_{k}^{*}u_{k}^{T}$$ (20)

#### 3.1.3 Evaluation methods. (1)GRA-TOPSIS method

The relative distance of GRA-TOPSIS scientifically reflects the closeness of this scheme to the ideal scheme, which has shown this method is more effective than GRA or TOPSIS methods [21]. In this paper, the GRA-TOPSIS method is used to reflect P, L, E subsystem development degree comprehensively.


$$U_{i}=\frac{D_{i}^{+}}{D_{i}^{+}+D_{i}^{-}}=\frac{\alpha y_{i}^{-}+\beta r_{i}^{+}}{\alpha {(y}_{i}^{+}+y_{i}^{-})+\beta {(r}_{i}^{+}+r_{i}^{-})};(\alpha +\beta =1)$$

$$\begin{array}{c} y_{i}^{+}=\sqrt{\sum_{j}w_{j}{\left(z_{ijt}-z_{j}^{+}\right)}^{2}},\\ y_{i}^{-}=\sqrt{\sum_{j}w_{j}{\left(z_{ijt}-z_{j}^{-}\right)}^{2}},\\ r_{i}^{+}=\frac{1}{n}\sum_{j}^{n}\left(\frac{\min|z_{j}^{+}-z_{ijt}|+\varepsilon \max|z_{j}^{+}-z_{ijt}|}{\left|z_{j}^{+}-z_{ijt}|+\varepsilon \max|z_{j}^{+}-z_{ijt}\right|}\right),\\ r_{i}^{-}=\frac{1}{n}\sum_{j}^{n}\left(\frac{\min|z_{j}^{-}-z_{ijt}|+\varepsilon \max|z_{j}^{-}-z_{ijt}|}{\left|z_{j}^{-}-z_{ijt}|+\varepsilon \max|z_{j}^{-}-z_{ijt}\right|}\right). \end{array}\}$$ (21)

Where, $y_{i}^{+},y_{i}^{-}$ stand for the positive and negative ideal solution of Euclid distance, $r_{i}^{+},r_{i}^{-}$ stand for the positive and negative ideal solution of the grey correlation degree distance. Whereas, *α*, *β* is the weight and usually set to *α* = *β* = 0.5. Respectively, $z_{j}^{+},z_{j}^{-}$ stand for the maximum and minimum value of the weighted evaluation index. *ε* is the resolution coefficient and generally set to 0.5. The PLE system(U)contains production resilience subsystem (*U*_1_), living resilience subsystem (*U*_2_), and ecological resilience subsystem (*U*_3_).

#### (2)CCDM method

Coupling refers to the system of interactions, influences, and relationships from *U*_1_ to *U*_*m*_, formula can be simplified to Eq (22) in two subsystem and Eq (23) among three subsystems:

$$C={\left\{(U_{1}∙U_{2})/{\left[\left(U_{1}+U_{2}\right)\right]}^{2}\right\}}^{\frac{1}{2}}$$ (22)

$$C={\left\{(U_{1}∙U_{2}∙U_{3})/{\left[\left(U_{1}{+U_{2}+U}_{3}\right)\right]}^{3}\right\}}^{\frac{1}{3}}$$ (23)

The coupling coordination degree model can be calculated as follows, which reflects the consistency and coordination degree of the system development.


$$D=\sqrt{C*T};T=\alpha U_{1}+\beta U_{2}$$ (24)

$$D=\sqrt{C*T};T=\alpha U_{1}+\beta U_{2}+\varphi U_{3}$$ (25)

Whereas *α*, *β*, *φ* denote undetermined coefficients, define *α* = *β* = *φ* and sum to 1. In two subsystems, whereas *α*, *β* denote 1/2 in this study. Referring to existing papers [14], Table 3 shows the development level classification, to express the urban resilience and subsystem development level briefly and clearly.

**Table 3:** The classification of development level.

Calculation Results	Classification	Performance characteristic
0.00–0.20	Strong imbalance	Mutual functional restriction causes serious imbalance and significant contradictions.
0.21–0.40	Moderate imbalance	Mutual functional constraints, lead to imbalance and contradiction
0.41–0.50	Basic coordination	Basic coordination between functions and normal development.
0.51–0.80	Moderate coordination	Mutual functional promotion and eventual orderly and sound development.
0.81–1.00	Strong coordination	All functions develop in a highly coordinated and orderly way, resulting in a high level of mutual promotion and development.

#### (3) ODM method

In order to better formulate strategies and suggestions to promote urban resilience, this article uses the ODM to obtain the degree of obstacles and their factors that constrain urban resilience, providing targeted decision-making basis for local urban development [23].


$$Z_{ij}=\frac{F_{ij}\times W_{ij}}{\sum_{i=1}^{n}\left(F_{ij}W_{ij}\right)}\times 100\%;F_{ij}=1-x_{ij}$$ (26)

Where *Z*_*ij*_ is the obstacle degree, *F*_*ij*_ represents the deviation degree of the indicators, *W*_*ij*_ represents the factor contribution degree, with the weights of the evaluation indices directly adopted in this study.

### 3.2 Data acquisition and processing

Sichuan province is located between 97°21’-108°31’E and 26°03’-34°19’N, with a length of 1075 kilometers from east to west and a width of 921 kilometers from north to south. It is bordered by 7 provinces (autonomous regions and municipalities), Qinghai, Gansu and Shaanxi in the north, Chongqing in the east, Yunnan and Guizhou in the south, and Tibet in the west. It is an important intersection and transportation corridor that connects South China and Central China, connects Southwest and Northwest, and communicates Central Asia, South Asia and Southeast Asia. The landform of Sichuan is complex, with mountains as the main feature, with four landform types: mountains, hills, plains and plateaus, accounting for 74.2%, 10.3%, 8.2% and 7.3% of the province’s area, respectively. Sichuan province is one of the most prone to geological disasters in the country, due to the different factors of climate, topography, geological structure and so on. There are four major regions and 18 prefecture level cities, including western of Sichuan (Chengdu, Deyang, Ziyang, Ya’an), eastern of Sichuan (Suining, Nanchong, Dazhou, Bazhong), southern of Sichuan(Meishan, Panzhihua, Leshan, Neijiang, Zigong, Yibin, Luzhou), and northern of Sichuan(Mianyang, Guangyuan, Guang’an).

Statistical data used in this paper were obtained from the China City Statistical Yearbook (2012–2022) (National Bureau of Statistics), and the Sichuan Statistical Yearbook (2012–2022) (Sichuan Provincial Bureau of Statistics and Survey Office of the National Bureau of Statistics in Sichuan). After standardizing the original data (2011–2021), the processing steps for data with results as follows.

Step 1: Based on the PLE index system in Table 1 to calculate indicator weights. The FAHP hierarchical model structure is divided into subsystem layer, criterion layer, and primary indicator layer, so as to use Eqs (1)–(9) to determine the weight based on FAHP matrix of each level. Firstly, compare the importance of the different criterion layers in the same subsystem separately, and next compare the importance of different indicators for the same criterion layer, and finally weighting is used to determine the importance of indicators for the subsystem. In EM method, the data (2011–2021) describing the 25 indicators for 18 cities were processed using Eqs (12)–(15). Blend weights are processed and shown in Table 4. The weight of the P,L,E subsystem’s indicators is a value between 0 and 1, which refers to the importance of an indicator relative to the subsystems. The greater the data value, the greater the importance, and respectively the total weight of production resilience, living resilience, ecological resilience subsystem is 1 under FAHP, EM, and FAHP-EM.

**Table 4:** Weights of subsystem indicators.

Goal	Indicator	EM	FAHP	FAHP-EM
P subsystem	X1	0.19	0.15	0.18
	X2	0.22	0.11	0.19
	X3	0.05	0.12	0.07
	X4	0.05	0.14	0.08
	X5	0.06	0.09	0.07
	X6	0.03	0.19	0.08
	X7	0.06	0.11	0.07
	X8	0.33	0.09	0.26
L subsystem	X9	0.02	0.11	0.05
	X10	0.23	0.14	0.20
	X11	0.07	0.09	0.07
	X12	0.11	0.06	0.09
	X13	0.07	0.13	0.09
	X14	0.30	0.10	0.23
	X15	0.07	0.11	0.08
	X16	0.01	0.09	0.04
	X17	0.08	0.08	0.08
	X18	0.02	0.04	0.03
	X19	0.03	0.05	0.04
E subsystem	X20	0.04	0.25	0.11
	X21	0.10	0.31	0.17
	X22	0.39	0.10	0.30
	X23	0.42	0.07	0.31
	X24	0.02	0.15	0.06
	X25	0.02	0.12	0.05

Step 2: The GRA-TOPSIS method is used to calculate three subsystems development level. From 2011 to 2021, the P,L,E subsystem development level of 18 cities in Sichuan are shown in Table 5.

**Table 5:** P,L,E subsystem’s development level.

City	P subsystem				L subsystem				E subsystem			
	2021	2011	growth rate and it’s ranks		2021	2011	growth rate and it’s ranks		2021	2011	growth rate and it’s ranks	
Chengdu	0.73	0.39	87.18%	1	0.64	0.49	30.61%	1	0.62	0.49	26.53%	10
Zigong	0.37	0.29	27.59%	13	0.42	0.33	27.27%	2	0.52	0.40	30.00%	7
Panzhihua	0.43	0.35	22.86%	17	0.42	0.36	16.67%	16	0.60	0.39	53.85%	2
Luzhou	0.38	0.28	35.71%	8	0.44	0.35	25.71%	6	0.44	0.38	15.79%	15
Deyang	0.44	0.31	41.94%	3	0.42	0.36	16.67%	16	0.44	0.38	15.79%	15
Mianyang	0.52	0.33	57.58%	2	0.46	0.39	17.95%	14	0.45	0.39	15.38%	17
Guangyuan	0.33	0.25	32.00%	10	0.44	0.37	18.92%	13	0.48	0.36	33.33%	3
Suining	0.35	0.25	40.00%	5	0.42	0.33	27.27%	2	0.45	0.37	21.62%	13
Neijiang	0.33	0.27	22.22%	18	0.42	0.33	27.27%	2	0.46	0.35	31.43%	4
Leshan	0.37	0.29	27.59%	13	0.43	0.37	16.22%	18	0.46	0.35	31.43%	4
Nanchong	0.33	0.24	37.50%	6	0.49	0.41	19.51%	11	0.47	0.36	30.56%	6
Meishan	0.36	0.27	33.33%	9	0.41	0.35	17.14%	15	0.44	0.36	22.22%	12
Yibin	0.41	0.29	41.38%	4	0.45	0.36	25.00%	9	0.48	0.39	23.08%	11
Guang’an	0.33	0.26	26.92%	15	0.43	0.34	26.47%	5	0.45	0.42	7.14%	18
Dazhou	0.33	0.24	37.50%	6	0.46	0.38	21.05%	10	0.45	0.35	28.57%	8
Ya’an	0.36	0.29	24.14%	16	0.44	0.35	25.71%	6	0.47	0.37	27.03%	9
Bazhong	0.30	0.23	30.43%	11	0.44	0.35	25.71%	6	0.45	0.29	55.17%	1
Ziyang	0.32	0.25	28.00%	12	0.43	0.36	19.44%	12	0.43	0.37	16.22%	14
Average value	0.39	0.28	39.29%		0.45	0.37	21.62%		0.48	0.38	26.53%	

Step 3: Use the CCDM method with the P,L,E subsystem development level above, the PLE system’s coupling coordination degree, that is the urban resilience development level, can be determined as shown in Table 6.

**Table 6:** Urban resilience development level.

City	2021	2020	2019	2018	2017	2016	2015	2014	2013	2012	2011
Chengdu	0.47	0.46	0.46	0.45	0.43	0.42	0.41	0.41	0.41	0.39	0.39
Zigong	0.38	0.37	0.37	0.37	0.36	0.35	0.35	0.35	0.34	0.34	0.33
Panzhihua	0.40	0.38	0.38	0.38	0.38	0.38	0.37	0.37	0.37	0.35	0.35
Luzhou	0.37	0.37	0.37	0.36	0.36	0.35	0.35	0.34	0.34	0.34	0.33
Deyang	0.38	0.37	0.37	0.37	0.36	0.35	0.35	0.35	0.35	0.34	0.34
Mianyang	0.40	0.39	0.39	0.39	0.37	0.37	0.36	0.36	0.36	0.35	0.35
Guangyuan	0.37	0.36	0.36	0.35	0.35	0.34	0.34	0.34	0.33	0.33	0.33
Suining	0.37	0.36	0.36	0.36	0.35	0.35	0.34	0.33	0.33	0.32	0.32
Neijiang	0.37	0.36	0.36	0.35	0.34	0.34	0.33	0.33	0.33	0.32	0.32
Leshan	0.37	0.37	0.36	0.36	0.35	0.35	0.34	0.34	0.34	0.34	0.33
Nanchong	0.38	0.37	0.37	0.36	0.36	0.35	0.35	0.34	0.34	0.34	0.33
Meishan	0.36	0.36	0.36	0.35	0.35	0.34	0.34	0.33	0.34	0.33	0.33
Yibin	0.39	0.37	0.37	0.36	0.36	0.35	0.34	0.34	0.34	0.34	0.34
Guang’an	0.37	0.36	0.36	0.36	0.36	0.35	0.35	0.35	0.34	0.34	0.33
Dazhou	0.37	0.36	0.35	0.35	0.34	0.34	0.34	0.33	0.33	0.32	0.32
Ya’an	0.38	0.37	0.36	0.35	0.35	0.35	0.35	0.34	0.34	0.34	0.33
Bazhong	0.36	0.36	0.35	0.34	0.35	0.34	0.33	0.33	0.33	0.32	0.31
Ziyang	0.36	0.36	0.36	0.35	0.35	0.34	0.34	0.33	0.33	0.33	0.32
Average value	0.38	0.37	0.37	0.36	0.36	0.35	0.35	0.35	0.34	0.34	0.33

**Fig 2:**
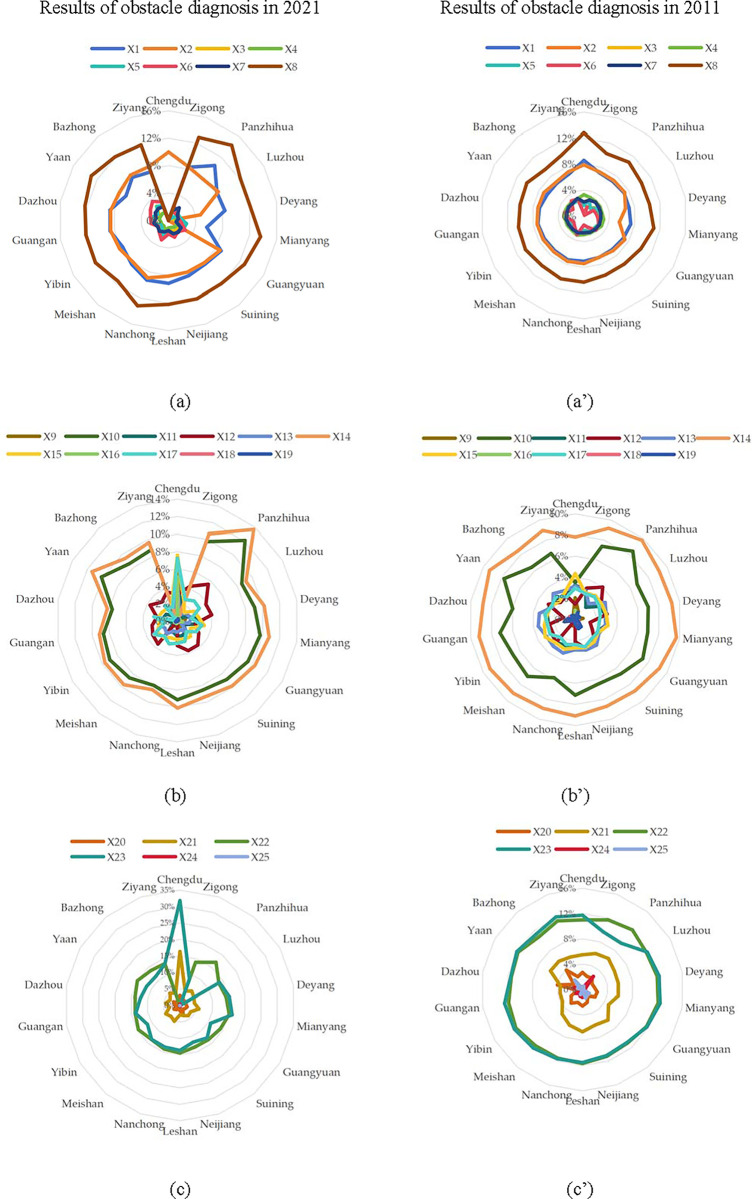
The results of factor obstacle diagnosis.

**Fig 3:**
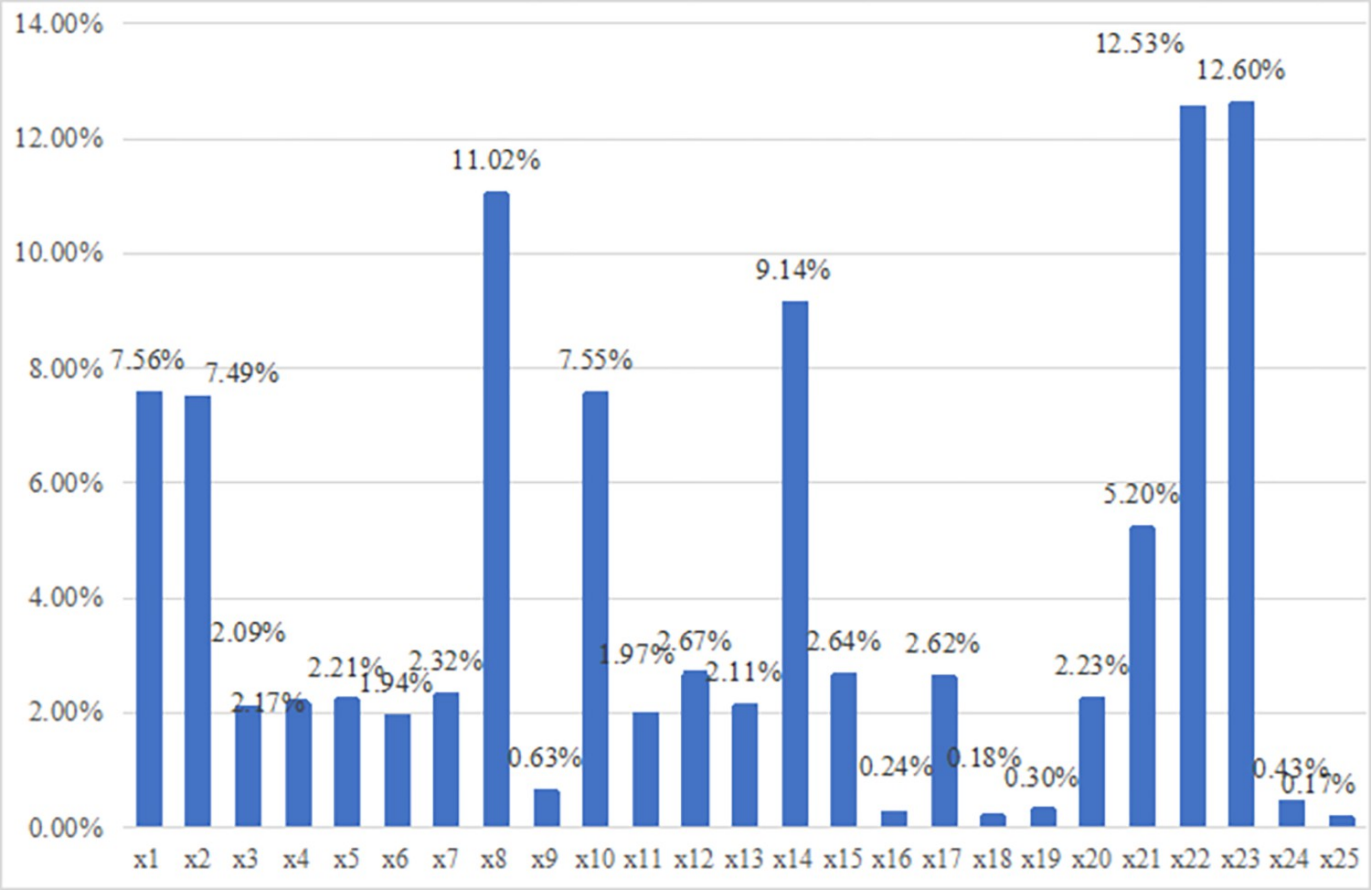
The factor contribution degree.

Step 4: According to the data processing methods and mathematical models given previously in Eq (26), the factor contribution degrees of indicators are calculated in Fig 2. The numerical size represents the contribution to the urban resilience system coupling coordination degree. In order to facilitate the search for important indicators, the average factor obstacle diagnosis degree of each indicator is calculated in Fig 3.

## 4 Results and discussion

### The urban resilience level

Different cities have the different development rates in terms of urban resilience development. As Table 6 shows the urban resilience system development level from 2011 to 2021, most cities represent at the moderate imbalance level, as well as basically maintained at this level, but Chengdu (the capital of Sichuan Province) is reaching the basic coordination degree level since in 2013. Table 7 shows the detailed relative ranking changes of 18 cities from 2011 to 2021. Obviously, Chengdu, Panzhihua, Mianyang and Yibin have always at the forefront and maintain the comparative advantage. In addition, Zigong, Luzhou, Deyang, Guangyuan, Leshan, Nanchong, Guang’an, Ya’an, Bazhong and Ziyang have relative slightly ranking changes, and the variation range is from 1 to 3. However, Suining, Neijiang, Dazhou, and Meishan have changed significantly, and variations are even beyond 5. The maximum variation value of Meishan even reached 10. From different regions, the urban resilience levels present the trend of regional contiguous development, it can be observed that the development of cities in the East Sichuan region is relatively increasing, while the development of cities in the North Sichuan region is relatively lagging behind.

**Table 7:** The relative ranking of urban resilience level from 2011 to 2021.

Region	City	Rankingin 2021	Ranking in2011	Changes
West Sichuan	Chengdu	1	1	−
	Deyang	5	4	↓1
	Ziyang	16	14	↓2
	Ya’an	5	6	↑ 1
East Sichuan	Suining	9	14	↑ 5
	Nanchong	5	6	↑ 1
	Dazhou	9	14	↑ 5
	Bazhong	16	18	↑ 2
South Sichuan	Meishan	16	6	↓10
	Panzhihua	2	2	−
	Leshan	9	6	↓3
	Neijiang	9	14	↓5
	Zigong	5	6	↑ 1
	Yibin	4	4	−
	Luzhou	9	6	↓3
North Sichuan	Mianyang	2	2	−
	Guangyuan	9	6	↓3
	Guang’an	9	6	↓3

Note: ↑ stands for the ranking increase, *−*means no change, ↓ stands for the ranking decrease and the number 0–10 indicates the amount of increase/decrease.

**Fig 4:**
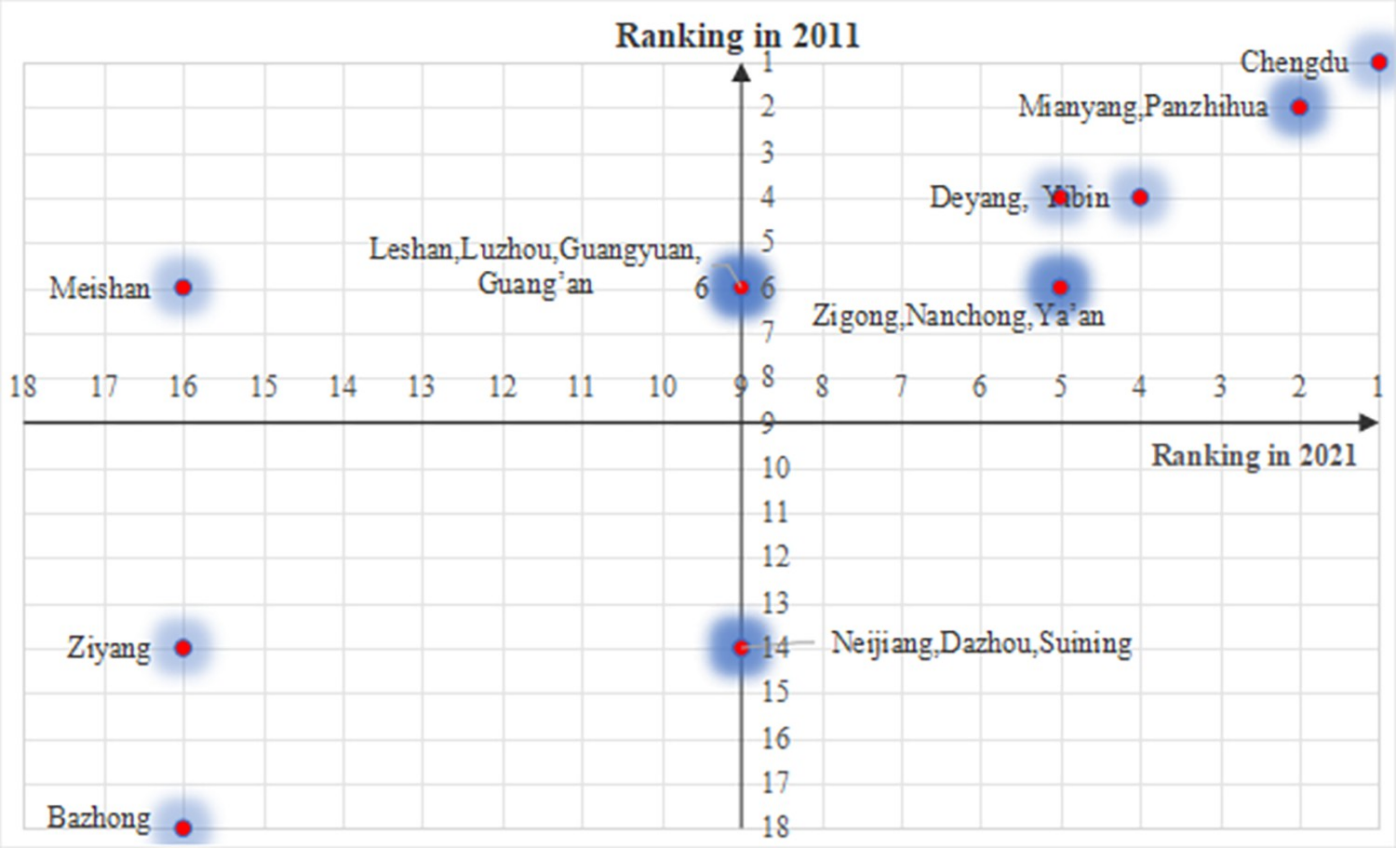
The relative ranking of urban resilience level.

Fig 4 is a quadrant chart composed of the ranking of urban resilience levels in 2011 and 2021,18 cities in Sichuan province can be divided into four different types. The first type is high development foundation and high development level, it includes Chengdu, Mianyang, Panzhihua, Deyang, Yibin, Zigong, Nanchong and Ya’an. The second type is poor development foundation and high development level, it includes Neijiang, Dazhou and Suining. The third type is high development foundation and poor development level, it includes Leshan, Meishan, Luzhou, Guangyuan and Guang’an. The four type is poor development foundation and poor development level, it includes Ziyang and Bazhong.

**Fig 5:**
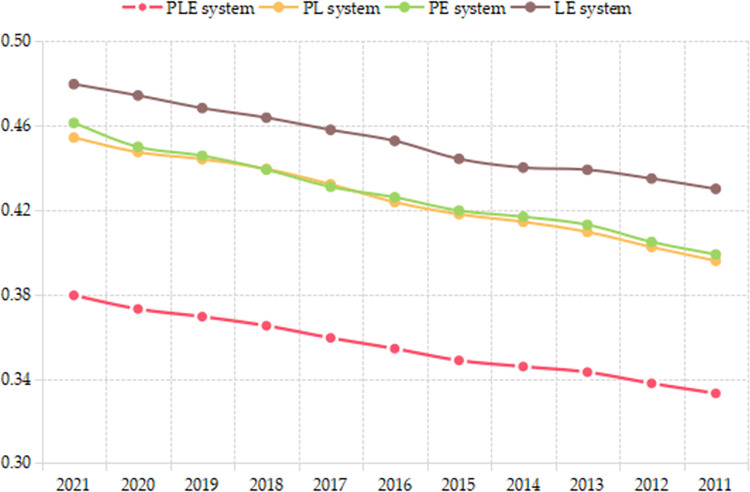
The development level of PLE, PL, PE, LE system.

All cities will still face great improve pressure, and P, L, E subsystem is the important guarantee for the formation of coupling coordination and interaction for urban resilience. By using the average development level of 18 cities, Fig 5 shows a general change trend for PLE, PL, PE, LE system from 2011 to 2021. The PLE system development level is generally falling behind than two subsystem’s coupling coordination development, such as PL, PE, LE system, which shows that the coupling and coordination effect of the urban resilience is not strong enough.

### The subsystem development level

As Table 5 shows, with the development of economy and society from 2011 to 2021, the P,L,E subsystem’s development level shows a growing change trend, and the growth rate changes of each subsystem and city are different. Such as Chengdu, Mianyang and Deyang have played a good leading role in P subsystem, and the development level far exceeds other cities in Sichuan Province from 2011 to 2021. For the L subsystem, Chengdu, Zigong, Suining and Neijiang have relatively good rankings in terms of growth rate changes. In addition, Bazhong, Panzhihua and Guangyuan have the good growth rates in the E subsystem.

According to average growth rate values of the P,E,L subsystem are 39.29%, 21.62%, and 26.53%, respectively. Correspondingly, the P subsystem’s growth change>the E subsystem’s growth change >the L subsystem’s growth change, which indicates that production policy is effective and requires continuous promotion, as well as it’s urgent to strengthen measures of ecological resilience and life resilience. By comparing the growth rates of P, L, E subsystems in 18 cities, it can find out the weak subsystems (Table 8) and corresponding development suggestions can be provided.

**Table 8:** The weak subsystems of 18 cities.

City	The weak subsystems	City	The weak subsystems
Bazhong	P	Ya’an	P,E
Neijiang	P	Guang’an	P,E
Zigong	P	Panzhihua	P,L
Chengdu	E	Leshan	P,L
Suining	E	Guangyuan	P,L
Yibin	E	Deyang	L,E
Luzhou	E	Dazhou	L,E
Nanchong	L	Meishan	L,E
Ziyang	P,L,E	Mianyang	L,E

### Indicators factor contribution analysis

As shown in Fig 2, the factors influencing the urban resilience development level are different with varying proportions over time, but the main obstacles in most cities are similar. For P subsystem, the most prominent obstacle factors are x8(total export-import per capita), x2(per capita of R&D spending), x1(per capita local financial expenditure on science and technology). However, with the achievements and advantages in Chengdu’s import and export economy, the relative main obstacle indicator of urban resilience development has become x2(per capita of R&D spending); For L subsystem, the most prominent obstacle factors are x14(number of people with basic medical insurance), x10(the number of employees). What’s special is that these indicators are no longer obstacle indicators for Chengdu, and its biggest obstacle indicator has become x15(per capita expenditure on social security and employment). For E subsystem, the most prominent obstacle factors are x21(per capita park green area), x22(length of urban drainage pipeline), x23(number of public toilets per person). For Chengdu, along with the obstacle factor of x22(length of urban drainage pipeline) has been well adjusted and reduced, and the proportion of the largest obstacle factor x23(number of public toilets per person) has undergone significant changes.

The P,L,E subsystem’s average factor obstacle diagnosis degree of 18 cities is 36.80%, 30.04%, and 33.16% respectively. It can be seen the P subsystem, is an important guarantee for the formation of coupling coordination and interaction for urban resilience. From the factor level as seen in Fig 3, the most prominent obstacle factors are x1(per capita local financial expenditure on science and technology), x2(per capita of R&D spending), x8(total export-import per capita), x14(number of people with basic medical insurance), x22(length of urban drainage pipeline), x23(number of public toilets per person) and the contribution values reach 7.56%,7.49%, 11.02%, 9.14%,12.53%, 12.60% respectively. This indicates that these obstacle indicators play a crucial role in the process of system coupling and collaborative development. If these indicators can be improved, the overall level of urban resilience collaborative development will be further enhanced.

Enhancing urban resilience is an important issue in the current sustainable development of cities. For the P subsystem, is an important guarantee for the formation of coupling coordination and interaction for urban resilience. Technology is the primary productive force, and its key is to increase investment in scientific and technological innovation and transform innovative achievements. In addition, the L subsystem should increase the pilot of basic medical insurance for urban residents and the full coverage of new rural cooperative medical care, as well as improve the coverage rate of basic medical facilities, to achieve the goal of everyone enjoying basic medical security. Moreover, the E subsystem should focus on continuously enhancing the ecological environment of human settlements, and comprehensively enhancing urban awareness of disaster prevention and reduction.

## 5 Conclusions

This study proposed a comprehensive evaluation of urban resilience, selected 18 cities of Sichuan as the research subjects, and analyzed its integration level of urban resilience by using CCDM method from 2011 to 2021. With respect to the multidimensional coordinated development perspective of production resilience, living resilience, and ecological resilience factors to build a systematic indicator system in the urban resilience. Referred to the urban resilience level from 2011 to 2021 in 18 cities, most cities are at the moderate imbalance and basically maintained at this level, thus all cities will still face great improve pressure. In general, the PLE system development level is generally falling behind in other system’s coupling coordination development, which shows that the coupling and coordination effect of the urban resilience is far from enough.

It is urgent for the government and relevant stakeholders to take feasible measures for achieving a better coordination development level in Sichuan. Consequently, the detailed reference suggestions and effective measures put forwarded for policy makers and planners to promote urban resilience in Western China. The following are some detailed suggestions:(1) the government should increase financial investment to support urban infrastructure construction and renovation, and improve the resilience level of urban infrastructure. In addition, promoting the construction of smart cities by utilizing technologies such as big data and the Internet of Things to improve urban operational efficiency and emergency response capabilities.(2)the enterprises should improve the safety production level of the enterprise itself and reduce the risk of accidents, strengthen cooperation with government, community and other stakeholders to jointly address urban disaster risks, as well as implement corporate social responsibility and pay attention to the safety and well-being of employees, consumers, and community residents.(3)the community should strengthen the construction of community organizations and improve the ability of community self-management and self-service. Meanwhile, carrying out disaster risk education and training to enhance residents’ awareness and skills in disaster prevention and reduction. In addition, a community emergency plan is established to clarify the responsibilities and tasks of all stakeholders. (4) the citizens should enhance awareness of disaster prevention and reduction, pay attention to personal safety and changes in the surrounding environment. Simultaneously, citizens should actively participate in community disaster prevention and reduction activities, and pay attention to urban infrastructure construction and actively participate in urban planning and construction.

This research utilizes coupling coordination degree to evaluate urban resilience level and identifies the key factors, so as to promote the urban resilience development. The results of this research could widen the research fields and for the strategy decision of urban resilience in other areas in China as well as other countries. However, due to missing data, it cannot cover the situation of Aba Prefecture, Liangshan Prefecture, and Ganzi Prefecture in Sichuan Province. Therefore, further research can be conducted on urban resilience in these ethnic regions, which can comprehensively evaluate the overall situation of Sichuan Province and provide targeted suggestions for the development of urban resilience in ethnic regions.

## Supporting information

S1 Table P subsystem development level. (DOCX)

S2 Table L subsystem development level. (DOCX)

S3 Table E subsystem development level. (DOCX)

S1 File Data & methods. (XLS)
