# Supplementary material for: Assessing urban resilience based on production-living-ecological system using degree of coupling coordination: A case of Sichuan
Source: PLoS One. 2024 May 23;19(5):e0304002. doi: 10.1371/journal.pone.0304002 (PMC11115233; doi:10.1371/journal.pone.0304002)
Supplement: S2 Table — (DOCX) [file pone.0304002.s002.docx]

**S2 Table L subsystem development level.**

| **City** | **2021** | **2020** | **2019** | **2018** | **2017** | **2016** | **2015** | **2014** | **2013** | **2012** | **2011** |
| --- | --- | --- | --- | --- | --- | --- | --- | --- | --- | --- | --- |
| Chengdu | 0.64 | 0.64 | 0.64 | 0.65 | 0.61 | 0.59 | 0.53 | 0.52 | 0.51 | 0.50 | 0.49 |
| Zigong | 0.42 | 0.42 | 0.40 | 0.42 | 0.38 | 0.37 | 0.36 | 0.35 | 0.34 | 0.33 | 0.33 |
| Panzhihua | 0.42 | 0.41 | 0.41 | 0.40 | 0.39 | 0.39 | 0.39 | 0.39 | 0.38 | 0.38 | 0.36 |
| Luzhou | 0.44 | 0.44 | 0.43 | 0.42 | 0.42 | 0.41 | 0.38 | 0.37 | 0.37 | 0.36 | 0.35 |
| Deyang | 0.42 | 0.42 | 0.41 | 0.40 | 0.40 | 0.37 | 0.37 | 0.36 | 0.36 | 0.36 | 0.36 |
| Mianyang | 0.46 | 0.46 | 0.46 | 0.45 | 0.43 | 0.42 | 0.41 | 0.41 | 0.41 | 0.40 | 0.39 |
| Guangyuan | 0.44 | 0.44 | 0.43 | 0.43 | 0.42 | 0.41 | 0.40 | 0.39 | 0.39 | 0.38 | 0.37 |
| Suining | 0.42 | 0.42 | 0.41 | 0.41 | 0.40 | 0.40 | 0.37 | 0.36 | 0.35 | 0.34 | 0.33 |
| Neijiang | 0.42 | 0.42 | 0.41 | 0.40 | 0.39 | 0.36 | 0.36 | 0.35 | 0.34 | 0.35 | 0.33 |
| Leshan | 0.43 | 0.43 | 0.42 | 0.42 | 0.41 | 0.41 | 0.39 | 0.38 | 0.38 | 0.37 | 0.37 |
| Nanchong | 0.49 | 0.50 | 0.48 | 0.48 | 0.48 | 0.44 | 0.43 | 0.42 | 0.42 | 0.42 | 0.41 |
| Meishan | 0.41 | 0.41 | 0.40 | 0.40 | 0.40 | 0.39 | 0.37 | 0.36 | 0.36 | 0.36 | 0.35 |
| Yibin | 0.45 | 0.45 | 0.44 | 0.43 | 0.42 | 0.41 | 0.38 | 0.37 | 0.37 | 0.37 | 0.36 |
| Guang'an | 0.43 | 0.44 | 0.43 | 0.43 | 0.42 | 0.38 | 0.38 | 0.38 | 0.36 | 0.36 | 0.34 |
| Dazhou | 0.46 | 0.45 | 0.45 | 0.44 | 0.43 | 0.39 | 0.39 | 0.38 | 0.38 | 0.37 | 0.38 |
| Ya'an | 0.44 | 0.43 | 0.41 | 0.40 | 0.40 | 0.39 | 0.39 | 0.38 | 0.38 | 0.36 | 0.35 |
| Bazhong | 0.44 | 0.44 | 0.42 | 0.40 | 0.42 | 0.39 | 0.37 | 0.37 | 0.36 | 0.36 | 0.35 |
| Ziyang | 0.43 | 0.42 | 0.41 | 0.41 | 0.40 | 0.38 | 0.39 | 0.38 | 0.37 | 0.37 | 0.36 |
| Average value | 0.45 | 0.45 | 0.44 | 0.43 | 0.42 | 0.41 | 0.39 | 0.38 | 0.38 | 0.37 | 0.37 |
